# Functional foods and cancer on Pinterest and PubMed: myths and science

**DOI:** 10.4155/fsoa-2018-0023

**Published:** 2018-08-09

**Authors:** Graça Justo, Eloy Macchiute de Oliveira, Claudia Jurberg

**Affiliations:** 1 Biochemistry Department of the Institute of Biology, Roberto Alcântara Gomes at the Universidade do Estado do Rio de Janeiro, Rio de Janeiro, 20511-010, Brazil; 2 DataSUS, Health Ministry, Rio de Janeiro, 20031-142, Brazil; 3 Brazil Institute of Medical Biochemistry Leopoldo de Meis, Universidade Federal do Rio de Janeiro and Oswaldo Cruz Institute, Fundação Oswaldo Cruz. Rio de Janeiro, 21941-902, Brazil

**Keywords:** cancer, food, Pinterest, PubMed, social media

## Abstract

**Aim:**

This article examines whether social media postings dealing with cancer and so-called ‘functional foods’ simply reflect a fashionable subject or are based on scientific evidence.

**Methods:**

The first step consisted of an analysis of a sample of Pins published on Pinterest. The second consisted of an analysis to determine whether the content of the Pins was based on scientific research.

**Results:**

From a set of 507 Pins on cancer, we found 204 that also dealt with food. We selected 75 Pins representing different foods and we identified about 80,000 scientific articles on cancer and food indexed in PubMed.

**Conclusion:**

We concluded that material published on Pinterest has some correlation with the scientific literature.

## Lay abstract

The objective of this study was to identify the correlation between what Pinterest users share about food and cancer, and the scientific evidence on the relationship between cancer and consumption of specific foods. In order to do this, we used PubMed and studied 75 Pins published in Pinterest. We identified about 80,000 scientific articles in PubMed about cancer and those foods posted on Pinterest. We observed that the Pins published in Pinterest have some relation to scientific information despite it not being possible to establish if the correlation with the particular food and cancer involved prevention, cure or treatment.

The abundance of information available on theinternet, people's increasing dependence on theinternet for information [1], and the dissemination of information and content via social media platforms and services [2–4] has changed how people conduct their lives and relate to companies and institutions [5].

Brazil is the country with the fourth highest number of people connected to the internet, behind only PR China (731 million), India (462 million) and the United States (286 million). More than 120 million people in Brazil (50% of the population) have access to theinternet. Between 2000 and 2015 the number of people in Brazil with access to theinternet increased by 2,682% [6]. Brazilian users spend an average of 283 minutes per day on theinternet, more than any other nationality [7].

Social media has become so popular, both in Brazil and elsewhere, that experts [8,9] are already debating whether smartphone addiction – users becoming hooked on virtual and impersonal conversations to the detriment of a face-to-face dialogue – should be considered an  epidemic. In addition to the ultra-exposure caused by the internet, the desire for such inclusion has also become a compulsion in some cases. The Institute of Psychiatry at the University of Sao Paulo, Brazil [10] stated that in 2012, 25% of patients who sought care were addicted to social media. Currently, Brazilians spend more time on Facebook, Whatsapp, YouTube, Instagram and Pinterest than any other social media. Pinterest arrived in Brazil in 2012, and between 2015 and 2016 the number of users doubled [11]. Worldwide there are 175 million active Pinterest accounts [12].

Created in 2009 by Paul Sciarra, Ben Silbermann and Evan Sharp, the name Pinterest is a combination of the English words ‘pin’ and ‘interest’, and Pinterest quickly became one of the most successful online platforms, growing rapidly from 2010 onward. Pinterest offers its users – who are called ‘Pinners’ – a virtual pinboard on which they can post photos and text, called ‘Pins’. Pins can be saved in public or private folders and various forms of interaction with pinned content are possible: ‘likes’, comments, and sharing, known as ‘repinning’ [13,14]. Pinners usually focus on topics that interest them, posting material in a variety of forms and formats – stories, photographs, illustrations, diagrams, videos, reports and links. The platform serves as a filter for several other media, including traditional media [15] and other social media such as Facebook, YouTube and Instagram.

Another feature of the Pinterest network is the high level of sharing among users. According to Fisher [16], ‘the audience in social media is characterized as engaged, expressive and collaborative’.

This is precisely why the audience for social media is commonly referred to as ‘users’. The advent of social media revolutionized how audiences were viewed, because they moved from being passive consumers of content to actively engaged users. According to Bosker [17], Pinterest generates a high volume ofinternet traffic since more than 80% of the published content is made up of Repins, that is, cases in which a user republishes another user's content on his or her own panel. In addition, as Schmidt [14] explained, there are various ways in which Pinterest users can interact. A user can follow other users or follow topics of interest, receiving alerts when new content is published. Users can still send messages directly to other people on the platform and can invite other users interested in their content to follow them. Folders can also be created collaboratively, thus creating more traffic and engagement.

## Health & nutrition in Pinterest

Although it has already been described in the literature [18–20] as one of the main themes, food and beverages are neglected in Brazilian studies on social media, especially in Pinterest, despite all citizens’ interest in the internet, social media and cuisine.

Brazil is a country rich in natural resources and thanks to the tropical climate a wide range of Brazilian produce is available throughout the year. Brazilians have an appreciation of good food and, although they do not always adopt a healthy diet [21], they appreciate the importance of balanced nutrition [22].

Research carried out in the country [23,24] showed that Brazilians began to admire and appreciate food and cooking due to extensive coverage on television, in dedicated magazines and in traditional newspapers. Thus, the theme became ‘media consumption, endowed with visibility’ [24], and with power to transform the conception of Brazilians regarding food. Lavinas [24] had pointed out that 30 years ago the media did not allocate as much space to gastronomy as it does currently. When TV coverage of gastronomy first began to increase in the country it was mainly in the form of shows in which the presenters took viewers through specific recipes, step by step, but this changed as reality shows and other food-related programs began to appear. There was also a growth in interest in ‘functional foods’ and bioactive substances, mainly relating to the role of food in preventing or treating chronic diseases, and this was highlighted in a position paper published by the Academy of Nutrition and Dietetics [25].

The rise of the internet and social media has made food even more fascinating in the eyes of Brazilian users. We believe that it is of the utmost importance to follow developments in this virtual world, where messages of all kinds are rapidly proliferating [1,16] and to investigate whether information about food published on social media is scientifically accurate. Regan and colleagues [26] as well as other authors [27,28] pointed to the risk of news disclosure by social media. In their opinion, this fact may cause inaccurate or sensationalist information through unregulated communication area, unlike advertisement or traditional media, both regulated. Despite this, it is also possible to envisage social media as a potential positive source to spread accurate information. The authors reflect about the increased use of social media. Therefore, it is necessary to discuss how important it is to explore new opportunities for communication and how they can be used, avoiding risks. This issue becomes extremely relevant when it is considered that there are about 50 billion Pins related to food only on Pinterest.

Brazilians have changed their eating habits in recent years and the importance of healthy eating is now recognized [22]. Nevertheless there is still a need for greater awareness of the dietary risk factors associated with certain illnesses such as cardiovascular diseases and cancer [29].

## Food & cancer

Global estimates [30] suggest that 5–10% of all cancers are associated with genetic factors and the remaining 90–95% are closely linked to lifestyle factors, including smoking, diet, alcohol, sedentary lifestyle, obesity, pollutants and sun exposure [31]. More than two decades ago, a joint report by World Cancer Research Control and the American Institute for Cancer Research [32] argued that dietary modifications, together with the abolition of smoking, would reduce the overall incidence of cancers. Sugimura [33] stated that one third of cancer cases are linked to food and that food and additives are an important determinant of the incidence of cancer in several countries.

It is well known that not smoking, not drinking, controlling weight, eating well and taking regular physical exercise help to prevent cancer [34], but modern life can make it difficult to follow this advice. For example, people now have less time available for meal preparation and this has contributed to an epidemic of stress and obesity, among other negative health outcomes [34]. A review carried out almost 30 years ago [35] concluded that a quarter of the population had a diet low in fruit and vegetables and therefore had double the risk of developing cancer of those who had a rich diet in fruit and vegetables.

Later Vainio and Weiderpass [36] reviewed epidemiological studies on the possible cancer–preventive effects of fruit and vegetable consumption. They sought to quantify the effect that high or low fruit and vegetable consumption had on risk of developing each type of cancer. They concluded that there was only limited evidence that consumption of vegetables reduced the risk of developing cancers of the mouth, pharynx, esophagus, stomach, colon, larynx, lung and ovary. The evidence that fruit consumption had a preventive effect on bladder cancer was also limited. The authors did state, however, that consumption of fruit and vegetables might reduce the risk of developing other cancers and that the preventive effect varied across the world. More recent studies [37,38] still question whether the consumption of specific foods can protect against specific types of cancer. Perhaps in the future, these doubts about functional foods and bioactive substances still persist, but adequate and accessible information will never cease to be a precious commodity for any citizen.

The use of social media is widespread and due to the popularity of food-related content, users of social media see information about food on a daily basis. False information spreads widely and very rapidly and while the scientific community continues to debate the efficacy of consuming certain fruits and vegetables as a cancer prevention strategy, we believe that studies of how food and cancer are linked in social media could help to clarify what has aroused the interest of society and hence how the scientific community and communication professionals can disseminate accurate information most effectively. Therefore, we questioned whether the content of cancer-related food published in Pinterest was more a result of this being a fashionable topic or if it was based on scientific evidence.

## Goal

Our goal was to investigate the correlation between what Pinterest users share about food and cancer and the scientific evidence on the relationship between cancer and consumption of specific foods. To do this, we used the US National Library of Medicine run by the National Institutes of Health (PubMed) and Pinterest. Pinterest is an open medium on which anyone can publish content, and so it can be used to disseminate beliefs and information that are without any scientific foundation. By the same token it can also be used to disseminate scientific information. However, when we have cancer on the agenda setting [39], the border between reliable information and inaccurate health information could be tenuous, and could also provoke unnecessary fear or panic.The Agenda Setting Theory may determine what society debates, discusses and thinks about, since the mass media forces attention to certain issues [39]. In this study, we wanted to describe how the relationship between food and cancer is portrayed on Pinterest and to assess the extent to which the information disseminated via Pinterest is consistent with published scientific research.

## Methods

### Study design

This was a qualitative–quantitative study [40], divided into two phases. The first consisted of an analysis of a sample of Pins published in panel format on Pinterest, in Portuguese, focusing on the themes of food and cancer. The second consisted of analysis to determine whether the content of the Pins analyzed in the first phase was based on scientific research.

The data for the first phase of the research were Pins published on panels on the Pinterest platform [15] in a private folder built for this research. We focused our research on the Pins themselves, so we did not investigate the links in each of them. The analysis of Pins was based on the method proposed by Bardin [41], who recommended starting with a preanalytical reading of all the material and then defining the objectives of the analysis and a guiding question.

The first stage was to collect a sample of Pins. We accessed the Pinterest platform and entered the word ‘cancer’ as search term at the social network search engine. During January, February and March of 2017, we performed frequent searches. All Pins were saved and the process was only halted when the content retrieved became repetitive.

Bardin [41] termed the set of documents that will be submitted to analytical procedures the ‘corpus’. Our initial corpus was the collection of cancer-related Pins published on Pinterest between January and March 2017, from this corpus we selected those dealing with food. All kinds of food were considered, independent of being processed or not, or being genetically modified, or even having other food constituents or additives.

This material was then organized into an Excel (Microsoft, WA, USA) table to provide an overview of the content. Next, the entire corpus was read carefully to allow us to identify its characteristics.

The Excel table contained detailed information about the Pins: food cited; compound cited; properties claimed (cure, treatment, prevention and risk factor) types of cancer (skin, mouth, breast, ovary, leukemia, stomach); engagement (numbers of ‘likes’, comments, Repins); web address; any reference to a scientific journal.

We then refined the coding of the corpus in order to identify its important characteristics [41] and attempted to interpret the results.

Based on the result of the first phase of the research, we accessed the PubMed platform, during April, May, June and July of 2017. We started by searching titles and summaries for names of foods, beverages and ingredients (translated into English) which had appeared in the Pin corpus combined with the word ‘cancer’. A new Excel table was created for the data collected in this phase. We tried to find out the scientific name, the total number of articles published and the number of manuscripts published in the last 5 and 10 years; how many referred to *in vivo* and *in vitro* experiments; how many bibliographic reviews have been made in the last 5 and  10 years.

After this, we selected the 10 foods that generated the greatest engagement in Pinterest and did a further search in PubMed. This time the search strings were as follows: ‘[food] AND cancer AND (prevention OR cure OR treatment). All the titles, abstracts and links retrieved constituted the second corpus, which was also read and analyzed.

## Results & discussion

In the first stage of the research, carried out on the social media platform Pinterest, a search for posts related to ‘cancer’ retrieved 507 Pins in Portuguese. These formed the initial corpus for the research [41]. A notable finding was that a great variety of foods were mentioned in posts with the subject ‘cancer’. We therefore decided to evaluate the correlation between all these food and compounds and the disease. From the 507 Pins, 204 were about cancer and food; these were saved in a private folder for subsequent analysis. There were a number of Pins presenting the same kind of food. We discarded the repeats and only 75 remained. We analyzed textual content and impact (links, ‘likes’ and comments). We selected those that presented, at the same time, links to other pages and mentioned different foods. We also determined which types of cancer were mentioned most frequently in these Pins and whether they were associated with diagnosis and suggested treatments; whether the food in question was claimed to prevent, cure or cause cancer; and whether the information was based on published scientific articles.

The 75 Pins were shared 26,992-times by other Pinners. No comments or ‘likes’ were associated with these Pins, which surprised us. Liking a post represents minimal engagement, because of the three available actions (likes, repins and comments) it is the simplest and quickest to execute. Sharing or Repinning is considered medium engagement, because the Pinterest user is adding the content to his or her own account. Commenting represents the highest level of engagement, because it requires the user to reflect on the topic in question and write about it, thus stating his or her opinion publicly [42].

The foods found to be associated with cancer included fruits, vegetables, proteins, carbohydrates, spices and beverages throughout the world, such as alcoholic drinks. Foods associated specifically with the Amazon region, such as açai, a fruit widely used in Brazilian cuisine in both food and drinks, were also mentioned (Table 1).

**Table 1.** **Foods linked to cancer in Pinterest.**

**Food**	**Cancer–related properties**				**Repins**

	***Cause***	***Preventive agent***	***Curative agent***	***Treatment***		
1) Saffron		1			33 (0.12)

2) Açai (*Euterpe oleracea*)		1			8 (<0.01)

3) Alcohol	1				1 (<0.01)

4) Lettuce		1			784 (2.9)

5) Garlic				1	157 (0.55)

6) Prune (in relation to colon cancer)		1			4 (<0.01)

7) Rice (in relation to lung cancer)	1				0 (<0.01)

8) Oats		1			784 (2.9)

9) Olive oil		1			784 (2.9)

10) Aloe vera/aloe vera syrup				1	116 (0.42)

11) Potato chips	1				1 (<0.01)

12) Beet		1			6 (<0.01)

13) Baking soda					7 (<0.01)

14) Broccoli		1			784 (2.9)

15) Coffee (in relation to breast cancer)	1				1 (<0.01)

16) Red meat	1				5 (<0.01)

17) Avocado pit		1			2400 (8.89)

18) Jaboticaba peel				1	1 (<0.01)

19) Brazil nut				1	6 (<0.01)

20) Carrot		1			784 (2.9)

21) Blackberry tea		1			1288 (4.77)

22) Ginger tea/ginger (ovary, prostate, liver and colon)			1		1361 (5.04)

23) Tea and juice of papaya leaves				1	970 (3.59)

24) Green tea (in relation to skin cancer)		1			57 (0.21)

25) Tea and juice of soursop leaves				1	1037 (3.84)

26) Mushrooms				1	1 (<0.01)

27) Cabbage		1			5 (0.01)

28) Dandelion				1	751 (2.78)

29) Spinach		1			3 (<0.01)

30) Soya extract (in relation to breast cancer)			1		4 (<0.01)

31) Fluorine	1				4 (<0.01)

32) Lemon tree leaf				1	2887 (10.69)

33) Red fruits		1			6 (<0.01)

34) Ginger		1			4 (<0.01)

35) Soursop				1	404 (1.49)

36) Janaúba (*Himatanthus drasticus*)					9 (<0.01)

37) Milk (in relation to breast and prostate cancer)	1				33 (0.12)

38) Soya milk (in relation to breast cancer)	1				7 (<0.01)

39) Lycopene (prostate)					7 (<0.01)

40) Lemon (in relation to skin, mouth, breast, stomach and colon cancer)					2 (<0.01)

41) Frozen lemon			1		183 (0.67)

42) Flaxseed			1		42 (0.15)

43) Absinthium			1		33 (0.12)

44) Honey with ginger				1	2703 (10.01)

45) São Caetano melon			1		937 (3.41)

46) Miso soup		1			7 (<0.02)

47) Strawberry		1			1 (<0.02)

48) Nut		1			4 (<0.01)

49) Apricot kernel oil		1			1 (<0.01)

50) Essential oils (in relation to colon cancer)				1	1020 (3.7)

51) Coconut oil				1	8 (0.02)

52) Hard–boiled eggs (in salad)		1			8 (0.02)

53) White bread (in relation to lung cancer)	1				0 (<0.01)

54) Fish	1	1			785 (2.9)

55) Pepper		2			12 (0.04)

56) Cheese				1	10 (0.03)

57) Horseradish				1	1 (<0.01)

58) Cabbage (in relation to colon cancer)				1	527 (1.95)

59) Canned cabbage		1			17 (0.06)

60) Pomegranate (breast)				2	332 (1.22)

61) Arugula		1			1 (<0.01)

62) Salt (in relation to stomach cancer)	1				1 (<0.01)

63) Pumpkin seed (in relation to prostate cancer)		1			9 (0.03)

64) Soya				1	1 (<0.01)

65) Tomato soup and saffron		1			98 (0.36)

66) Carrot, cucumber and kale juice		1			794 (2.9)

67) Chia juice		1			1313 (4.8)

68) Soursop leaf juice				1	1000 (3.7)

69) Orange and flaxseed juice (in relation to colon cancer)		1			1 (<0.01)

70) Apple juice (in relation to colon cancer)				1	47 (0.17)

71) Tamarind		1			0 (<0.01)

72) Tomato		1			784 (2.9)

73) Grape		1			784 (2.9)

74) Raisin		1			19 (0.07)

75) Vitamin C				1	3 (<0.01)

Total	11	35	6	22	26,992

We identified two foods linked to skin cancer (green tea and lemon); one linked to mouth cancer (lemon); six to breast cancer (coffee, soya extract, milk, soya milk, lemon and pomegranate); one to ovarian cancer (ginger); two to stomach cancer (lemon and salt); four to prostate cancer (ginger, milk, lycopene, and pumpkin seed); two to lung cancer (bread and rice); one to liver cancer (ginger); and seven to colon cancer (plum, ginger, lemon, coconut oil, horseradish, flaxseed and apple juice).

35 of the 75 Pins (46%) referred to foods and ingredients important in tumor prevention; 22 (29%) of these were designed as adjuvants in the treatment of cancer; 11 (14%) were described as causing cancer; and only six (8%) as foods that cured the disease [Table 1]. We found only one Pin (on absinthe) referring to a scientific periodical. So we asked ourselves whether there was any scientific evidence linking the foods to cancer or whether this social media content was without factual foundation.

We addressed this question in the second stage of the research, by searching for scientific articles on the 75 foods related to cancer in Pinterest in PubMed. The search was carried out between May and July 2017 and identified 79,317 scientific articles (Table 2). First of all, we carried out separate searches for each of the 75 foods combined with the word ‘cancer’. Then we carried out three further series of searches with the 10 foods that generated the most engagement in Pinterest; the foods were combined with the words ‘cancer’ and ‘prevention’ in one search, ‘cancer’ and ‘cure’ in the second, and ‘cancer’ and ‘treatment’ in the third.

**Table 2.** **Foods and compounds in PubMed.**

**Food**	**Scientific name**	**Number of articles**	***in vitro***	***in vivo***	**Reviews**	**Number of articles in the last 5 years**	**Number of articles in the last 10 years**	**Number of reviews in the last 5 years**	**Number of reviews in the last 10 years**
1) Saffron	–	160 (0.20%)	42	25	27	78	112	15	18

2) Açai	*Euterpe oleracea*	14 (0.02%)	3	1	1	7	14	1	0

3) Alcohol	–	12807 (16.15%)	181	86	2165	3907	6871	624	1104

4) Lettuce	–	80 (0.10%)	9	4	6	26	35	2	3

5) Garlic	–	993 (1.25%)	199	133	207	239	433	57	94

6) Prune	–	93 (0.12%)	10	7	15	29	56	3	9

7) Rice	–	3824 (4.82%)	345	306	368	1398	2070	128	200

8) Oats and cereals	–	21 (0.03%)	4	2	8	12	13	6	7

9) Olive oil	–	819 (1.03%)	88	73	162	250	421	55	96

10) Aloe vera/aloe vera syrup	–	347 (0.44%)	56	34	40	115	195	18	26

11) Canned potato chips	–	13 (0.02%)	0	1	0	4	8	0	0

12) Beet	*Beta vulgaris*	136 (0.17%)	21	15	9	39	62	5	7

13) Baking soda	Sodium bicarbonate	455 (0.57%)	50	34	48	94	159	11	15

14) Broccoli	–	1030 (1.30%)	167	134	152	276	518	46	80

15) Coffee	–	1667 (2.10%)	26	14	250	445	764	72	125

16) Red meat	–	1268 (1.60%)	29	19	258	498	812	101	162

17) Avocado seed	–	44 (0.06%)	11	4	5	14	27	1	3

18) Jaboticaba peel	*Myrciaria cauliflora*	2 (<0.01%)	0	0	0	1	1	0	0

19) Brazil nuts	–	14 (0.02%)	1	0	5	6	8	2	2

20) Carrot	–	143 (0.18%)	16	9	8	41	67	2	4

21) Blackberry leaf tea	–	1 (<0.01%)	0	0	0	1	1	0	0

22) Ginger tea	–	416 (0.52%)	84	44	77	202	304	31	49

23) Papaya leaf tea	–	1 (<0.01%)	1	0	0	0	1	0	0

24) Green tea	–	4351(5.48%)	767	528	817	1241	2310	233	438

25) Soursop leaf tea	–	0 (<0.01%)							

26) Mushrooms	–	961 (1.21%)	201	121	105	379	672	55	76

27) Cabbage	–	997 (1.26%)	121	91	115	270	497	34	61

28) Dandelion	–	33 (0.04%)	9	5	3	11	21	1	2

29) Spinach	–	139 (0.18%)	27	15	12	39	62	3	6

30) Soyabean extract	–	77 (0.10%)	14	13	13	20	40	4	8

31) Fluorine	–	333 (0.42%)	57	80	57	140	230	5	17

32) Lemon tree leaf	–	13 (0.02%)	1	0	2	5	10	0	0

33) Red fruits	–	665 (0.84%)	58	34	181	241	392	59	100

34) Ginger	*Zingiber officinale*	234 (0.30%)	25	29	9	57	100	1	2

35) Soursop	*A. muricata*	10 (<0.01%)	5	3	0	8(	10(	0	0

36) Janaúba	*Himatanthus drasticus*	0 (<0.01%)	1	0	0	1	1	0	0

37) Milk	–	204 (0.26%)	23	14	24	49	92	10	16

38) Soy milk	–	134 (0.17%)	12	8	23	22	63	6	9

39) Lycopene	–	1202 (1.52%)	140	89	288	268	547	70	138

40) Lemon	–	1300 (1.64%)	176	123	144	403	655	47	76

41) Frozen lemon	–	6 (<0.01%)	0	0	0	0	2	0	0

42) Flaxseed	–	300 (0.38%)	40	22	56	83	154	17	25

43) Losna or Absinthe	*Artemisia* sp.	272 (0.34%)	85	40	30	133	211	13	24

44) Honey and ginger	–	1 (<0.01%)	1	0	0	1	1	0	0

45) São Caetano melon	*Momordica charantia*	111 (0.14%)	37	24	7	41	70	4	6

46) Miso soup	–	34 (0.04%)	0	0	0	4	9	0	0

47) Strawberry	–	220 (0.28%)	29	20	33	47	85	9	18

48) Walnut	–	858 (1.08%)	63	46	170	319	528	61	101

49) Apricot kernel oil	–	0 (<0.01%)	0	0	0	0	0	0	0

50) Essential oils	–	443 (0.56%)	37	25	94	142	227	24	45

51) Coconut oil	–	99 (0.12%)	8	8	6	18	27	1	1

52) Boiled eggs (in the salad)	–	0 (<0.01%)	0	0	0	0	0	0	0

53) White bread	–	48 (0.06%)	1	0	1	8	18	0	1

54) Fish	–	19432 (24.50%)	1323	1163	2057	5670	9903	589	1002

55) Pepper	–	687 (0.87%)	103	56	98	236	394	30	53

56) Cheese (nisin)	Nisin	19 (0.02%)	4	4	2	12	14	2	2

57) Horseradish	–	1035 (1.30%)	137	79	22	204	335	2	3

58) Cabbage	–	302 (0.38%)	42	24	45	67	122	7	17

59) Canned cabbage	–	2 (<0.01%)	0	0	0	1	1	0	0

60) Pomegranate	–	222 (0.28%)	59	35	52	119	185	29	45

61) Arugula	–	3 (<0.01%)	2	0	0	2	2	0	0

62) Salt (salt and cancer risk)	–	2104 (2.65%)	43	30	420	995	1356	148	235

63) Salt	–	11644 (14.68%)	1360	893	1516	4123	5963	471	749

64) Pumpkin seeds	–	6 (<0.01%)	1	0	0	3	4	0	0

65) Soya	–	1104 (1.39%)	178	114	220	198	431	33	72

66) Tomato and saffron soup	–	0 (<0.01%)	0	0	0	0	0	0	0

67) Carrot, cucumber and kale juice	–	0 (<0.01%)	0	0	0	0	0	0	0

68) Chia juice	–	1253 (1.58%)	95	75	96	598	953	49	81

69) Soursop leaf juice	–	0 (<0.01%)	0	0	0	0	0	0	0

70) Orange and flaxseed juice	–	0 (<0.01%)	0	0	0	0	0	0	0

71) Apple juice	–	45 (0.06%)	15	9	5	16	31	1	4

72) Tomato	–	636 (0.80%)	87	72	119	171	301	26	51

73) Tamarind	*Tamarindus indica*	18 (0.02%)	4	5	0	10	13	0	0

74) Grape	–	850 (1.07%)	149	102	93	230	471	36	68

75) Raisin	–	13 (0.02%)	2	3	0	1	4	0	0

76) Vitamin C	Ascorbic acid	2559 (3.23%)	253	178	407	539	988	87	152

Total		79,317	7133	5122	11,153	24,819	41,447	3347	5708

## Prevention & cancer

A report produced by about 30 independent experts for the WHO and Food and Agriculture Organization (FAO) in 2003 warned of a rapid rise in chronic diseases, especially in developing countries, and noted that chronic diseases are responsible for 59% of deaths worldwide. This report strongly suggested that a diet low in saturated fats, sugar and salt, and rich in fruits and vegetables, combined with regular physical activity, would do much to reduce mortality due to chronic diseases. It is worth remembering that 90–95% of all cancers are associated with lifestyle factors, including obesity, diet and alcohol consumption factors [30,31]. Between 1974 and 1985 the proportion of people in Brazil who were overweight or obese was about 23%, but this had risen to 49% by 2008/2009. This makes the role of food in disease prevention an important topic.

An interesting finding from our analysis of Pins was that 35 foods were associated with cancer prevention (46% of all items associated with cancer in Pinterest). The PubMed search retrieved more than 30 thousand articles on these 35 foods - 40% of the total 79,317 articles.

Of these 35 Pins correlated to prevention, only in six cases (17%), which included nine foods – apricot, kernel oil, boiled eggs, tomato and safflower soup, carrot juice, cucumber juice and kale, and orange juice and flaxseed – no scientific article was found. Then, we tried to find out if there would be scientific literature in a search with just the name of these nine foods (apricot, eggs, tomato, saffron, carrot, cucumber, kale, orange and flax), regardless of the preparation mode, such as teas and soup. In this new search, 8876 scientific articles were found in PubMed.

## Treatment & cancer

The American Academy of Nutrition and Dietetics, which was founded in 1917 and is the largest institution in the field of nutrition, has stated [25] that functional foods may have health benefits when consumed as part of a varied diet and that there is scientific evidence of their benefits. The academy has stated that there is growing evidence that certain components of food can offer health benefits. Some of these compounds are even used in treatment and the number of scientific articles on their use has grown a lot in recent years.

23 of the 75 bioactive foods that were the subject of Pins were claimed to contain compounds that treated cancer. For these same foods, in a new search in PubMed, we found 7773 articles (9.7% of the total). A search for ‘soursopAND cancer’ (i.e., not specifying mode of preparation) retrieved 10 articles, eight of which were published in the last 5 years. Most of these articles were part of basic research indicating anti-tumor properties of soursop.

## Cancer & cure

The scientific community is very cautious about using the term ‘cure’ in relation to cancer. In oncology, it is effectively taboo and a 2013 survey found that approximately 80% of doctors were reluctant to use it, and would consider doing so only when a patient remained cancer-free 6–10 years after diagnosis [43].

Prasad [44] examined articles published between January and December 2012 on the Thompson Reuters Web of Science portal in an attempt to understand how the term ‘cure’ was used in articles dealing with cancer, and after some exclusion, the author identified that half of the 29 manuscripts used the word to affirm that the disease was incurable and approximately one third (34.5%) used the term ‘cured’ to refer to patients who had reached the point at which their survival rate was similar to a control group.

Perhaps the hesitation to apply with word ‘cure’ to cancer is responsible for the relatively small numbers of references to curative foods on Pinterest and in the literature found in PubMed.

We found that only six of the 75 foods (8%) mentioned in Pinterest were claimed to be curative, these were: ginger, soya extract, frozen lemon, linseed, absinthe and São Caetano melon. These six foods were mentioned in 786 out of 79,317 articles in PubMed, just 0.99% of the total. It is worth clarifying that we found scientific publications on all the foods that were described as curative on Pinterest, although the articles in PubMed tended to mention treatment or anti proliferative effects and not necessarily cure.

## Risk & cancer

The PubMed searches on the 11 foods and beverages (14% of the total of 75) identified as increasing cancer risk (alcohol, oats, potato chips, coffee, red meat, fluorine, milk, soya milk, white bread, fish and salt) retrieved 39,526 (49.8% of the total papers). It is noteworthy that in all cases the scientific articles also identified these foodstuffs as cancer risks.Our next step was to explore societal interest in foods identified as risk factors for cancer. We did this by looking at the level of engagement posts on these foods generated on Pinterest. Alcohol has been recognized by the WHO [44] as a cause of various cancers such as cancers of the oral cavity, pharynx, larynx and esophagus [45]. A joint International Agency for Research on Cancer (IARC) and WHO report [46] listing alcohol as a cause of liver, colorectal and breast cancers received little attention on Pinterest (a Pin with only one Repin). Alcohol consumption is very high in Brazil, even in young people, despite the prohibition on selling alcoholic beverages to children under 18 years of age [47].

Our next step was to explore societal interest in foods identified as risk factors for cancer. We did this by looking at the level of engagement posts on these foods generated on Pinterest. Alcohol has been recognized by the WHO [48] as a cause of various cancers such as cancers of the oral cavity, pharynx, larynx and esophagus [45]. A joint International Agency for Research on Cancer (IARC) and WHO report [46] listing alcohol as a cause of liver, colorectal and breast cancers received little attention on Pinterest (a Pin with only one Repin). Alcohol consumption is very high in Brazil, even in young people, despite the prohibition on selling alcoholic beverages to children under 18 years of age [47].

Table 3 shows the lack of interest, as indexed by the low number of Repins in Pinterest, in the other foods identified as risk factors.

**Table 3.** **PubMed versus Pinterest.**

**Compound**	**Number of articles in PubMed**	**Number of Repins on Pinterest**
Alcohol	73,000	1

Rice	3824	0

Canned potato chips	13	1

Coffee	1667	1

Red meat	1268	5

Fluorine	333	4

Milk	204	33

Soya milk	134	7

White bread	48	0

Fish	19,432	785

Salt	13,748	0

The 11 foods identified as risk factors for the development of cancer in the scientific literature available in PubMed and their representation in Pinterest.

Both alcohol and salt were mentioned in a large number of articles in PubMed (73,000 and 13,748, respectively) but this was related to their use in experiments rather than as foodstuffs. A further search using the search string ‘salt’ AND ‘cancer risk’ retrieved 2104 articles. Due to the large number of articles and in the absence of tools for analyzing them, we chose not to delve more deeply into the relationships of alcohol and salt with cancer both on social media and in the scientific literature.

## Subjects that are popular or heavily represented on Pinterest & PubMed

The next step involved investigating the ten foodstuffs that generated the highest level of engagement in the form of Repins, including lime leaf (2887 Repins), honey with ginger (2703), avocado core (2400), soursop tea or juice (2037), blackberry tea (1288), ginger tea (1361), chia juice (1313), essential oils (1,020), papaya (Carica papaya) leaf tea and juice (970) and São Caetano melon (937).

Three of these foodstuffs (avocado core, blackberry leaf tea and chia juice) were cited as preventing cancer, two (ginger and São Caetano melon) were claimed to be powerfully curative and five (soursop tea and juice, honey with ginger, papaya leaf juice and tea, lime leaf and oil of life) were described as treatments. None of the foods that generated high engagement were correlated with cancer risk.

Then, we opted to analyze the contents of these 10 Pins containing one or more foods with high performance from the number of Repins and their respective searches in PubMed (Table 4).

**Table 4.** **Number of articles retrieved from PubMed that mention the foods with high performance in Pinterest.**

**Foodstuff**	**Repins**	**PubMed**	**Reviews in the last 10 years**
Soursop	2037	10	0

Ginger	1361	396	46

Chia	1313	1253	81

Blackberry^†^	1288	123	13

Honey^‡^	2703	357	54

Citrus^§^	3072	1319	76

Essential oils	1020	443	45

Avocado	2400	44	3

Sao Caetano melon	937	111	6

Papaya	970	1	0

The relationship between mentions on Pinterest and mentions in the scientific literature, including reports of *in vivo* and *in vitro* research and bibliographic reviews.

^†^Blackberry tea. This result refers to the search only for the word ‘blackberry’.

^‡^Honey in association with ginger.

^§^This category includes the words ‘lemon’, ‘frozen lemon’ and ‘lime leaf’.

Among the most popular food Pins, lemon was cited as both a cure and a treatment, whilstpapaya and soursop leaves, honey with ginger and essential oils were described as treatments for cancer. Blackberry, according to Pinners, prevents cancer, and ginger is described as having curative powers.

We investigated these highly cited foodstuffs in more depth, by looking for evidence of their effectiveness in the form scientific publications indexed in PubMed in which they were associated with prevention, treatment or cure for cancer. We searched titles, abstracts and links for the following search string: ‘cancer AND [food] AND (prevention OR cure OR treatment) [Table 5]. All the articles retrieved were read to determine whether they were relevant. Table 5 shows the scientific literature on the foods most commonly mentioned on Pinterest, together with the claims made for their curative, preventive and therapeutic powers, including capacity to reduce the effects of chemotherapy or radiation therapy.

**Table 5.** **PubMed searches for each food with the keywords ‘prevention’, ‘treatment’ and ‘cure’.**

**Food**	**Prevention**	**Treatment**	**Cure**	**Reducing the effects of chemotherapy**	**Protects against radiation**
Soursop	2	0	0	0	0

Ginger^†^	4	63 (7^‡^)	3	4	1

Chia	3^§^	2	0	0	0

Blackberry	3	3	0	0	0

Honey	4	51 (5^¶^)	0	2	4

Lemon	1	7^#^	0	1	3

Essential oils	7	4	0	1	

Avocado	1	8	0	0	0

Melon of Sao Caetano	9	10	0		

Papaya	3	9	0	0	0

Representation of foods (regardless of preparation, e.g., tea, juice, or part of the plant involved, e.g., pulp; leaf; seed) that generated high Pinterest engagement in the scientific literature over the past 5 years.

^†^Articles in the last 5 years.

^‡^Reviews in the last 5 years.

^§^One of the articles suggests that it does not prevent cancer.

^¶^Reviews in the last 5 years and an article reporting that there is no evidence that it has anticancer properties.

^#^Two articles deal with fruit peel.

It is important to emphasize that these values are based exclusively on the results of a database search using the search string ‘[food] AND cancer AND (prevention OR treatment OR cure). It is surprising that some of the foods that attracted most interest amongst Pinners, such as papaya, posts on which generated more than 900 Repins, had attracted little academic interest. Pinterest users described papaya tea as a functional food but we retrieved only one article on papaya as a functional food from PubMed. The scientific paper associating papaya with cancer was by a Japanese group [49] and also cited papaya leaves as potentially useful for the treatment and prevention of cancer and other allergenic diseases. The authors went on to suggest that papaya could perhaps be used in a vaccine. This broad spectrum of activity as a biofunctional food brings us reflections on the importance of more studies. The search for articles linking cancer with ‘honey with ginger’ (the Pinterest citation) found only one article, by a Malaysian group [50], that sought to demonstrate, *in vitro*, the preventive effect of these foods on colon cancer.

Pins on blackberry generated 1288 Repins and our PubMed searches retrieved several articles that described the bioactive components of various berries (strawberry, raspberry, blueberry, blackberry and the Indian gooseberry) in general terms, as well as their antioxidant and anti-cancer activity. However, there were only two articles mentioning blackberry tea, one on its potential as an inhibitor of tumor cells in colon cancer [51] and another [52] on its anti-proliferative effect on tumor cells. This shows that further research is needed on this foodstuff and demonstrates that foodstuffs which have attracted interest from social media users for their cancer-related properties have not always been extensively researched. In our research on PubMed, we did not distinguish whether the cancer-related foods described in the articles mentioned cure, treatment or prevention.

Estimates suggest that at present there are about 600,000 new cases of cancer in Brazil every year [53]. The most frequent cancers – excluding nonmelanoma skin cancers – are prostate cancer (69,000 new cases p.a.), followed by breast cancer (57,000 new cases p.a.,) and colorectal cancer (33,000 new cases p.a.). Interestingly, the types of cancer most mentioned in this Pinterest research associated with food were exactly these three. There were seven Pins claiming that certain foodstuffs (plum, ginger tea, lemon, coconut oil, cabbage, orange, flaxseed and tamarind) had a beneficial effect on colon cancer. There were seven Pins linking breast cancer with foodstuffs (coffee, soya extract, fruit and vegetables, milk and soya milk, lemon and pomegranate). There were also four Pins linking prostate cancer with ginger, milk, lycopene and pumpkin seed.

### Open access deposition

The data from Pinterest and PubMed have been made freely available as a deposition on the ZENODO open access platform [54]. The deposition contains an abstract and two tables.

As a result, it is also relevant to discuss the impact of social media and health communication. As Freeman [55] mentioned, ‘new media have the potential to increase the accessibility of content, the amount of content and the number of people who can create and share that content’. There is no doubt, as mentioned by Moorhead and colleagues [56], that social media are a powerful tool, including both developed nations and developing countries such as Brazil. Although there are several benefits to the use of social media for health communication, the information exchanged should be monitored in both developed and developing nations, since we should not forget that social media have no filter [27]. This phenomenon increases the risk for incorrect information. In the case of ‘functional food’, food may be associated with both risks and benefits [27]. Red wine consumption, for example, has been reported various times as having beneficial health effects (e.g., on the cardiovascular system) as well as the negative health effects related to alcohol consumption. In the case of allergies, social media may spread knowledge of risks (e.g., milk for children with lactose intolerance). Another aspect that may be considered is that some of the food suggestions may produce interactions with conventional drugs used for treatment, furthermore, oftentimes patients do not inform physicians about their diet.

A positive topic to highlight is the fact pointed by Mythen [57] “*risk rumors may spread quickly online, and the interactive nature of social media presents a unique opportunity for inaccuracies to be challenged and corrected*”. In times of unverified information, there are no doubts about high social media engagement; at the same time, it is indisputable that other users can immediately change wrong post content. In Brazil, where social media users spend more time than any other nationality online [7], debates are intense.

## Final considerations

The aim of this research was to identify the foods, beverages and ingredients associated with cancer on one of Brazil's most popular social media, Pinterest, and to determine whether these associations were supported by scientific evidence, through PubMed searches. Our intention was to establish how trustworthy information on Pinterest about the cancer-related properties of foodstuffs is, as much of the material circulating online is not reliable.

We were able to measure the interest and engagement that each foodstuff generated on Pinterest and compare this with the level of interest from the research community, as indicated by a number of publications. Seventy-five out of 507 Pins on cancer (15%) were related to foodstuffs and these foodstuffs were mentioned in approximately 80,000 scientific articles in PubMed.

We conclude that there is overlap between the Pinterest content linking foods, beverages and ingredients to cancer and the scientific literature in PubMed, as about 90% of the foods cited on Pinterest were also mentioned in publication indexed in PubMed. The Pins linking foodstuffs and cancer were shared some 27,000-times in the form of Repins, which provides evidence that there is interest in foods that have been linked to cancer. However, Pinterest users seem reluctant to express opinions on this subject, as very few comments were found on our corpus of 75 food plus cancer Pins. We also remarked that Pinterest users do not bother to provide any scientific evidence for their claims about the cancer–related properties of foods. Only one Pin, on absinthe, cited a scientific periodical.

It is worth mentioning that only 11 of the corpus of 75 Pins (just under 15%) claimed that consumption of a foodstuff increased cancer risk and these foods mentioned were also described as risk to ingest in PubMed.

It is important to emphasize that only 10% of the foodstuffs mentioned in our corpus of Pins were not mentioned in scientific papers in PubMed, although in another 10% of the cases, we identified little relevant scientific literature. When foods were correlated to a type of cancer on the Pinterest platform, the majority of the types mentioned were exactly the most common types of cancer in Brazil.

In view of these results, we concluded that the content of cancer-related foods published in Pinterest could be considered as a source of information, although it was not possible to establish for each food researched in PubMed the exact correlation with cancer, whether it acts in prevention, cure or treatment. This is probably the result of most studies dealing with basic research and *in vitro* experiments.

We were surprised by these results as we thought the claims made on Pinterest would prove to be sensationalist and without scientific foundation and we suggest that deeper analysis of the correlation between information published on social media and in the scientific literature is required. We also consider it important that continuous effort is made to raise awareness of the importance of reliable information on health-related topics. Efforts must be made to raise public awareness of the need to ensure that claims related to health and medicine have a scientific basis.

Despite the fact that the results of this study appointed a correlation between the ‘functional foods’ popularization in social media and the scientific literature, we cannot forget that these new tools do not have a filter and it is not possible to minimize the risk of incorrect data disclosure.

## Future perspective

To verify how Pinterest can influence behavioral change, based on what is published about cancer and food, we are applying Social Cognitive Theory in order to understand users’ behavior. Future studies are important to better understand the social media impact on behavior change and how we can use these resources for better health communication.

**Summary points**

- Brazil is the country with the fourth highest number of people connected to theinternet.
- More than 120 million people in Brazil (50% of the population).
- Brazilians spend more time on Facebook, Whatsapp, YouTube, Instagram and Pinterest than any other social media.
- Pinterest arrived in Brazil only in 2012, and 4 years later the number of users doubled.
- One of the top topics on Pinterest is food, and most of this is associated as ‘functional food’.
- There is an interest growth in ‘functional foods’ and bioactive substances, mainly related to the role of food in preventing or treating chronic diseases as cancer.
- Despite all Brazilian citizens’ interest on theinternet, social media and cuisine, food and beverages are neglected in Brazilian studies on social media, especially on Pinterest.
- There is an association between ‘functional foods’ published on Pinterest and the scientific literature.
- We found that about 90% of the foods cited on Pinterest were also mentioned in publication indexed in PubMed.
- New media opened the chance to people create and share content. They have the potential to increase the accessibility of content and the amount of content. The problem is lack of filter.
